# Targeting LIPA with ERX-41 Induces ER Stress and Inhibits Tumor Progression in Inflammatory Breast Cancer

**DOI:** 10.3390/biom16030481

**Published:** 2026-03-23

**Authors:** Zenaida Fuentes, Gaurav Sharma, Bianca A. Romo, Rahul Gopalam, Khaled Mohamed Nassar, Paulina Ramirez, Nicole Mejia, Chia-Yuan Chen, Scott Elmore, Henry Neal, Harika Nagandla, Panneerdoss Subbarayalu, Uday P. Pratap, Christoforos Thomas, Jung-Mo Ahn, Ganesh V. Raj, Suryavathi Viswanadhapalli, Ratna K. Vadlamudi

**Affiliations:** 1 Department of Obstetrics and Gynecology, University of Texas Health San Antonio, San Antonio, TX 78229, USA; zenaida.fuentes@vanderbilt.edu (Z.F.); sharmag1@uthscsa.edu (G.S.); gopalam.rahul@gmail.com (R.G.); nassark@livemail.uthscsa.edu (K.M.N.); ramirezp6@uthscsa.edu (P.R.); pratap@uthscsa.edu (U.P.P.); 2 Department of Biology, University of the Incarnate Word, San Antonio, TX 78209, USA; baromo@uiwtx.edu; 3 Department of Chemistry and Biochemistry, University of Texas at Dallas, Richardson, TX 75080, USA; chiayuan.chen@utdallas.edu (C.-Y.C.); scott.elmore@utdallas.edu (S.E.); henry.neal@utdallas.edu (H.N.); jungmo.ahn@utdallas.edu (J.-M.A.); 4 Houston Methodist Neal Cancer Center, Houston Methodist Research Institute, Houston, TX 77030, USA; hnagandla@houstonmethodist.org (H.N.); cthomas3@houstonmethodist.org (C.T.); 5 Greehey Children’s Cancer Research Institute, University of Texas Health San Antonio, San Antonio, TX 78229, USA; subbarayalu@uthscsa.edu; 6 EtiraRx Inc., Dallas, TX 75247, USA; ganesh@etira.life; 7 Mays Cancer Center, University of Texas Health San Antonio, San Antonio, TX 78229, USA; 8 Audie L. Murphy Division, South Texas Veterans Health Care System, San Antonio, TX 78229, USA

**Keywords:** inflammatory breast cancer, breast cancer, ERX-41, LIPA, ER stress

## Abstract

Approximately 2–4% of all breast cancer cases are inflammatory breast cancer (IBC), an extremely rare and severe subtype of the disease. Current therapies, including chemotherapy, surgery, and radiotherapy, remain insufficient, underscoring the need for novel therapeutic approaches. IBC exhibits elevated basal endoplasmic reticulum (ER) stress, suggesting a potential vulnerability. We recently developed ERX-41, a small molecule that exacerbates ER stress in cancer cells by inhibiting the endoplasmic reticulum-localized function of Lysosomal acid lipase A (LIPA). Here, we evaluated the therapeutic potential of ERX-41 in IBC models. ERX-41 markedly reduced the viability of IBC cells and significantly impaired clonogenic survival while promoting apoptosis. The specificity of ERX-41 was confirmed using LIPA-knockdown and LIPA-knockout cells. RT-PCR-based assays revealed rapid induction of *XBP1* splicing within 6 h of treatment, and Western blot analyses demonstrated activation of ER stress markers including CHOP, PERK, and ATF4. In KPL4 xenografts, ERX-41 treatment significantly decreased tumor volume, accompanied by reduced proliferation and increased ER stress marker expression by IHC. Collectively, these findings identify LIPA as a therapeutically actionable vulnerability in IBC and establish ERX-41 as a potential drug for IBC.

## 1. Introduction

Inflammatory breast cancer (IBC) is a rare, highly aggressive breast cancer with rapid onset and early spread [1,2,3]. IBC tumors frequently lack estrogen receptor alpha (ERα) and express detectable levels of ERβ [4], and most patients do not benefit from endocrine therapy [1]. Despite multimodal treatment strategies, including chemotherapy, surgery, and radiotherapy, overall outcomes for IBC remain poor, and many patients experience early recurrence, therapy resistance, and a 5-year survival rate of only 40% [5,6]. New targeted therapies for treating IBC are urgently needed.

IBC displays extensive molecular dysregulation, with genomic profiling revealing a high frequency of clinically relevant alterations that drive its aggressive behavior [7]. Targeted sequencing further shows that IBC tumors harbor numerous somatic mutations in key signaling pathways, contributing to increased cellular stress and tumor progression [7,8]. Complementing these genomic findings, gene-expression studies have identified a distinct poor prognosis signature enriched for pathways involved in inflammation, stress responses, and tumor aggressiveness [9]. IBC displays high basal ER stress driven by its inflammatory, cytokine-rich microenvironment. While this chronic ER stress supports IBC cell survival, it also creates a distinct therapeutic vulnerability [10]. Together, these alterations suggest that IBC relies on stress-adapted signaling environments that may engage ER stress and UPR pathways to sustain its highly aggressive phenotype.

Cancer cells operate under persistent proteotoxic and metabolic stress due to rapid proliferation, oncogene activation, hypoxia, nutrient limitation, and sustained protein synthesis demands [11,12]. The ER is a specialized organelle essential for several cellular processes, particularly protein folding and maturation [13]. Because IBC cells proliferate rapidly, they require elevated protein synthesis, folding, and maturation. To cope with resulting ER stress, they activate the unfolded protein response (UPR) [14,15].

UPR signaling initially promotes survival through attenuation of global translation, induction of chaperones such as GRP78/HSPA5, enhancement of ER-associated degradation (ERAD), and metabolic rewiring [16,17]. However, when ER stress is severe or prolonged, the same pathways can trigger apoptosis through mediators including ATF4 and CHOP, among others [18,19]. Importantly, many tumor types, including aggressive BC subtypes such as IBC, exhibit elevated basal ER stress, suggesting that cancer cells exist near the threshold of ER proteostasis capacity. We hypothesized that further aggravation of ER stress in IBC cells may selectively overwhelm IBC cells and drive cell death while sparing normal tissues.

Recently, we developed ERX-41, a small-molecule therapeutic that binds and inhibits Lysosomal acid lipase A (LIPA) and induces ER stress selectively in cancer cells [20]. Unlike traditional metabolic inhibitors, ERX-41 promotes ER stress responses in a manner that rapidly activates core UPR signaling and drives apoptosis [20]. This unique mechanism provides a strong rationale for exploring ERX-41 as an anti-cancer agent in other BC types such as IBC that are highly dependent on ER adaptation mechanisms for survival.

This study investigated ERX-41’s therapeutic activity and its mechanism of action in IBC. ERX-41 exhibited nanomolar potency, effectively reduced the cell viability of IBC cells and decreased clonogenicity. We confirmed the target specificity using LIPA knockout models. Mechanistic studies confirmed activation of ER stress upon ERX-41 treatment. Using the KPL4 xenograft model, we validated the in vivo efficacy of ERX-41 on IBC. Collectively, our findings demonstrate that targeting LIPA with ERX-41 triggers robust ER stress signaling and suppresses IBC tumor progression, highlighting ERX-41 as a promising therapeutic option for IBC.

## 2. Materials and Methods

### 2.1. Cell Culture Methods and Reagents

SUM149 and KPL-4 cell lines were used to model IBC [4]. SUM149 cells are a triple-negative IBC subtype lacking ER, PR, and HER2 expression, whereas KPL-4 cells are ER-/PR- with HER2 overexpression. Both cell lines were maintained in DMEM/F12 supplemented with 1 µg/mL hydrocortisone, 5 µg/mL insulin, 2% Antibiotic/Antimycotic, 1% sodium pyruvate, and 5% FBS. Antibodies against p-eIF2α, PERK, CHOP, and ATF4 were obtained from Cell Signaling Technology. The Ki67 antibody (ab1667) was purchased from Abcam (Cambridge, MA, USA), and the LAL/LIPA antibody (sc-58374) was sourced from Santa Cruz Biotechnology (Dallas, TX, USA). ERX-41 was synthesized following the protocol described in our prior publication [20].

### 2.2. LIPA Knockdown and LIPA Knockout

IBC cells were transfected with LIPA-targeting siRNAs (20 nM; siLIPA-1: SASI_Hs01_00141713, ctggacttgagctgtgtacca; siLIPA-2: SASI_Hs02_00302146, gagagcattccggaatgggag) or a non-targeting control siRNA (Universal Negative Control #1, Sigma-Aldrich, St. Louis, MO, USA) using Lipofectamine RNAiMAX (Invitrogen, Waltham, MA, USA) as per the manufacturer’s protocol. LIPA-knockout IBC cells were generated by lentiviral transduction using a validated LIPA-targeting gRNA as previously described [20]. LIPA knockdown or knockout was confirmed using Western blotting.

### 2.3. Cell Viability, Clonogenicity, Apoptosis, and Invasion Assays

For cell viability assays, IBC cells (1000–2000 cells/well) were plated in 96-well plates and treated with ERX-41 for 5 days, and viability was measured using the MTT assay. Clonogenic potential was assessed by plating 500 cells/well and treating with ERX-41 or vehicle for 5 days; colonies were methanol-fixed, stained with 0.5% crystal violet, and quantified using the ImageJ Colony Area plugin (v1.54p). Apoptosis was measured after 48 h of ERX-41 or vehicle treatment using Annexin V/PI staining (BioLegend #640914, San Diego, CA, USA) and by caspase-3/7 activity (Caspase-Glo^®^ 3/7, Promega Corporation, Madison, WI, USA) as described [21]. Cell invasion was evaluated using Matrigel-coated transwell inserts (8 µm, Corning, Glendale, AZ, USA) via Boyden chamber assays as described [21].

### 2.4. Western Blotting

Cells were lysed in RIPA buffer containing EDTA and Halt Protease/Phosphatase Inhibitor (Thermo Scientific, Waltham, MA, USA). Proteins were separated by 10–15% SDS-PAGE, transferred to nitrocellulose or PVDF membranes, probed with primary and secondary antibodies, and visualized using ECL substrates (Bio-Rad, Hercules, CA, USA). Western blot original images can be found in Supplementary Materials.

### 2.5. RT-qPCR

Cells were treated with vehicle or ERX-41 (100 nM) for 6 h, and RNA was extracted using TRIZOL. cDNA was generated with the High-Capacity cDNA Reverse Transcription Kit, and qRT-PCR was performed using PowerUp SYBR Green Master Mix under standard conditions with a 60 °C annealing temperature. All reagents were purchased from Applied Biosystems (Waltham, MA, USA). Primer sequences are provided in Table S1.

### 2.6. XBP1 mRNA Splicing Assay

Total RNA was isolated using the RNeasy Mini Kit. First-strand cDNA was synthesized with the SuperScript III First-Strand Synthesis Kit (Invitrogen, Waltham, Massachusetts, USA). To assess the relative levels of unspliced and spliced *XBP1*, semi-quantitative RT-PCR was performed using PCR SuperMix (Invitrogen, Waltham, MA, USA). *XBP1u*/*XBP1s* cDNA fragments were amplified by PCR using the primers listed below: *XBP1u*/*XBP1s*-F-5′CCTGGTTGCTGAAGAGGAGG3′ and *XBP1u*/*XBP1s*-R-5′CCATGGGGAGATGTTCTGGAG3′. GAPDH was used as a loading control, with primers as follows: 5′-GGGTCAGAAGGATTCCTATG-3′ and 5′-GGTCTCAAACATGATCTGGG-3′. PCR products were resolved on a 3% agarose gel.

### 2.7. Transmission Electron Microscopy (TEM) Studies

IBC cells treated with vehicle or ERX-41 (100 nM, 8 h) were harvested, fixed in 4% formaldehyde/1% glutaraldehyde, post-fixed in 2% OsO_4_, dehydrated in ethanol, and embedded in Poly/Bed^®^ 812. Ultrathin sections were cut, stained with uranyl acetate and lead citrate, and imaged using a JEOL JEM-1400 transmission electron microscope. ER cisternae area (mm^2^) was quantified using ImageJ [22].

### 2.8. In Vivo Xenograft Studies

All animal experiments were conducted in accordance with UT Health San Antonio IACUC-approved protocols and guidelines. The study was not blinded; investigators were aware of group allocations during assignment, experimental conduct, outcome assessment, and data analysis. KPL4 tumor xenografts were generated in the mammary fat pad of female SCID mice as previously described [21]. After tumor establishment, mice were randomly assigned to receive either the vehicle control or ERX-41 (10 mg/kg, intraperitoneally) for 5 days per week over a 40-day period, formulated in 0.3% hydroxypropyl cellulose as described [20]. A total of 8 animals were used in the study, with 4 animals assigned to the control group and 4 to the treatment group. Each animal contributed two tumors, resulting in *n* = 8 tumors per group for all analyses. Tumor growth was measured twice weekly using digital calipers, and volumes were calculated as ½ × (L × W^2^), where L is the longitudinal diameter and W is the transverse diameter. To minimize potential confounding factors, all animals were maintained under identical environmental and husbandry conditions, and all measurements were performed using the same procedures and equipment. No inclusion or exclusion criteria were applied. All animals and experimental units enrolled at the start of the study were included throughout the experiment, and no data points were excluded during data collection or analysis. At the end of the treatment period, mice were euthanized, and tumors were collected and processed for histological analysis.

### 2.9. Immunohistochemistry

Sections were blocked, incubated overnight with Ki-67 (Abcam 16667; 1:50) and GRP78 (#3177; 1:50, Cell Signaling Technology, Inc. Danvers, MA, USA) antibodies, then treated with the ImmPRESS^®^ HRP reagent for 30 min. Staining was developed with DAB (Vector Laboratories, Inc. Newark, CA, USA) and counterstained with hematoxylin. Ki-67 positivity and GRP78 H-scores were quantified from five random 20× fields.

### 2.10. Statistical Analysis

Data was analyzed using GraphPad Prism 10. Statistical significance was assessed using ANOVA or *t*-tests, with *p* < 0.05 considered significant. Because the H-score represents an ordinal variable, group comparisons were performed using the Kolmogorov–Smirnov (K-S) test, a nonparametric method that compares cumulative distributions without assuming normality or linear spacing between score categories.

## 3. Results

### 3.1. ERX-41 Suppresses IBC Cell Viability, Clonogenic Survival, Apoptosis Resistance, and Invasive Potential

**Figure 1:**
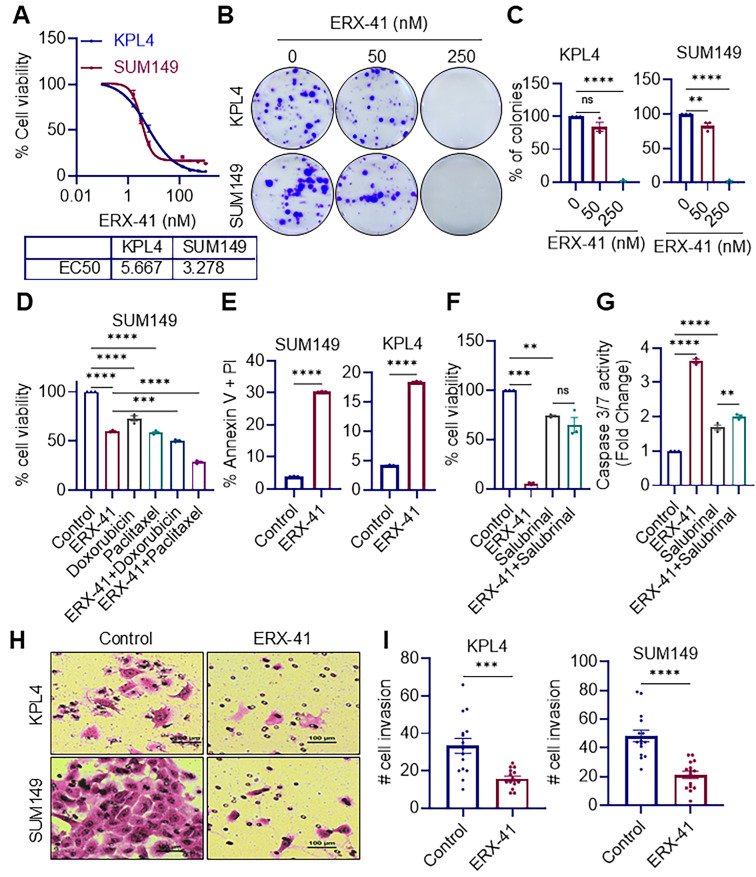
ERX-41 reduces viability and colony growth, triggers apoptosis, and suppresses invasion in IBC cell lines. (**A**) Dose–response curves showing the effect of ERX-41 on cell viability in KPL4 and SUM149 cells. Cells were treated with ERX-41 for 5 days, and viability was assessed using the MTT assay. (**B**) Representative images of colonies formed by KPL4 and SUM149 cells after treatment with ERX-41. (**C**) Bar graphs depicting the percentage of colonies formed by KPL4 and SUM149 cells under different ERX-41 concentrations. (**D**) SUM149 cells were treated for 72 h with ERX-41 (100 nM), doxorubicin (5 nM), paclitaxel (2.5 nM), or the indicated combination treatments. Cell viability was measured using MTT assay and normalized to untreated controls. (**E**) Quantification of apoptotic cells in SUM149 and KPL4 lines after ERX-41 (500 nM-24 h) treatment compared to control. (**F**,**G**) ERX-41-induced loss of cell viability is rescued by the ER stress inhibitor Salubrinal, whereas caspase-3/7 activation is partially suppressed. SUM149 cells were treated with ERX-41 (250 nM), Salubrinal (20 μM), or a combination of ERX-41 with Salubrinal for 48 h. Cell viability (**F**) and caspase-3/7 activity (**G**) were quantified and reported as fold change relative to control. (**H**) Representative microscopic images of invading KPL4 and SUM149 cells through Matrigel-coated transwell inserts after ERX-41 (250 nM-22 h) treatment versus control. Scale bar = 100 μm. (**I**) Bar graphs showing number of invaded cells in KPL4 and SUM149 lines after ERX-41 treatment compared to control. n = 3; data shown as mean ± SEM; ** *p* < 0.01, *** *p* < 0.001, **** *p* < 0.0001, ns = not significant.

To evaluate ERX-41’s therapeutic efficacy in IBC, we examined its effects on growth and survival using two well-established IBC models. ERX-41 treatment markedly reduced the viability of KPL4 and SUM149 cells as measured by MTT assays (Figure 1A). Further, ERX-41 profoundly inhibited long-term clonogenic growth in clonogenicity assays in both KPL4 and SUM149 cells (Figure 1B,C). Treatment of SUM149 cells with ERX-41 decreased viability relative to vehicle, indicating single-agent activity. Doxorubicin and paclitaxel also reduced viability compared with control. Notably, combined treatment with ERX-41 and either doxorubicin or paclitaxel produced significant decreases in viability, exceeding the effects of the respective single agents. Together, these data suggest that ERX-41 potentiates chemotherapy-induced cytotoxicity in SUM149 cells (Figure 1D). Annexin V/PI staining demonstrated a robust increase in apoptotic populations in both SUM149 and KPL4 cells following ERX-41 treatment compared to control (Figure 1E). To determine whether Salubrinal, an inhibitor of eIF2α dephosphorylation that reduces ER stress-induced cell death [23], could mitigate ERX-41-induced apoptosis, cell viability and caspase-3/7 activity were measured in SUM149 cells following treatment with ERX-41 alone or in combination with Salubrinal. Treatment with ERX-41 markedly decreased cell viability, reducing it to approximately one-third of control levels. Salubrinal alone moderately reduced viability, but when combined with ERX-41, it rescued the ERX-41-induced loss of viability, bringing the signal closer to baseline and eliminating the significant difference between the ERX-41 + Salubrinal and Salubrinal-alone conditions (Figure 1F). ERX-41 also robustly activated caspase-3/7, producing more than a three-fold increase over control. Although Salubrinal alone modestly increased caspase activity, co-treatment substantially attenuated ERX-41-mediated caspase-3/7 activation (Figure 1G). Together, these findings indicate that ER stress/UPR activation is functionally required for ERX-41-induced cell death. Given the aggressive and metastatic phenotype of IBC, we tested whether ERX-41 impacts invasive behavior. Matrigel invasion assays showed substantial reductions in the invasive capacity of both KPL4 and SUM149 cells upon ERX-41 treatment (Figure 1H,I). Collectively, these results demonstrate that ERX-41 inhibits IBC cell survival, reduces clonogenic potential, suppresses invasion *in vitro* and promotes apoptosis.

### 3.2. ERX-41 Triggers the Unfolded Protein Response and Activates ER Stress Signaling in IBC Cells

**Figure 2:**
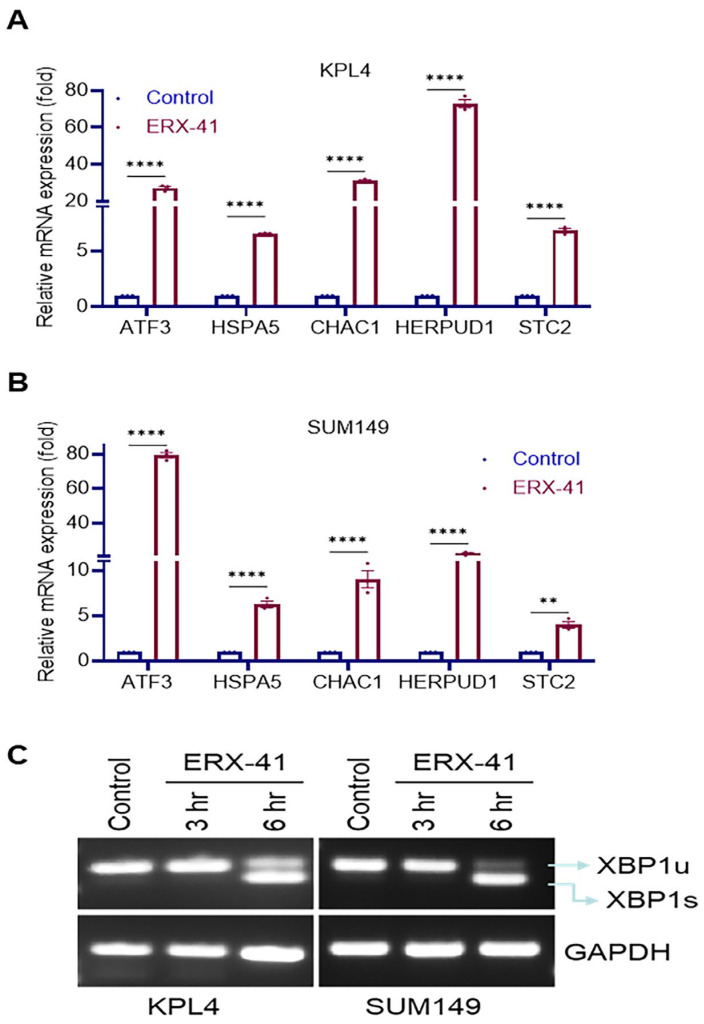
ERX-41 induces ER stress in IBC cells. (**A**,**B**) Relative mRNA expression levels of *ATF3*, *HSPA5*, *CHAC1*, *HERPUD1*, and *STC2* in KPL4 (**A**) and SUM149 (**B**) cells treated with ERX-41 compared to control. (**C**) Representative gel images showing XBP1 splicing (*XBP1u* = unspliced, *XBP1s* = spliced) in KPL4 and SUM149 cells after ERX-41 treatment for 3 and 6 h. GAPDH served as a loading control. n = 3; data represent mean ± SEM; ** *p* < 0.01; **** *p* < 0.0001.

ERX-41 was developed as a LIPA-targeting small molecule that promotes ER stress. We therefore tested whether ERX-41 treatment promotes activation of canonical markers of ER stress and UPR in IBC cells. RT-qPCR analyses revealed marked upregulation of multiple ER stress/UPR-responsive genes, including *ATF3*, *HSPA5 (GRP78)*, *CHAC1*, *HERPUD1*, and *STC2*, in both KPL4 and SUM149 cells following ERX-41 treatment (Figure 2A,B). To specifically evaluate activation of the IRE1α pathway, we conducted assays measuring X-box binding protein 1 (*XBP1*) splicing. ERX-41 induced robust *XBP1* mRNA splicing in both IBC models, detectable at as early as 3 h and stronger by 6 h post-treatment, consistent with rapid activation of ER stress signaling (Figure 2C).

### 3.3. ERX-41 Causes ER Ultrastructural Dilation and Activates PERK–eIF2α–ATF4/CHOP Signaling

**Figure 3:**
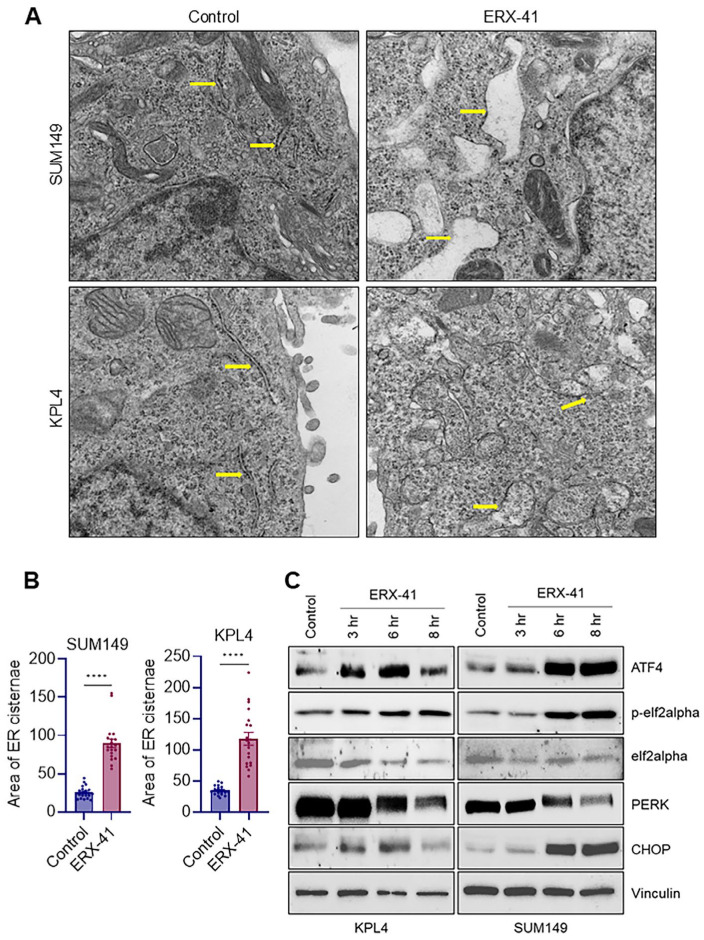
ERX-41 treatment results in prominent ultrastructural changes accompanied by increased expression of ER stress markers in KPL4 and SUM149 cells. (**A**) Transmission electron microscopy (TEM) images of SUM149 and KPL4 cells treated with ERX-41 or vehicle control. Yellow arrows indicate dilated endoplasmic reticulum structures following ERX-41 treatment. (**B**) Quantification of the area of endoplasmic reticulum cisternae (mm^2^, n = 20). (**C**) Immunoblot analysis of ER stress markers ATF4, phosphorylated eIF2α (p-eIF2α), eIF2α, PERK and CHOP in KPL4 and SUM149 cells after ERX-41 treatment for 3, 6, and 8 h. Vinculin was used as a loading control. **** *p* < 0.0001.

To determine whether ERX-41 induces structural ER stress phenotypes, we performed transmission electron microscopy (TEM). Compared to control cells, ERX-41-treated SUM149 and KPL4 cells exhibited clear dilation of ER cisternae, consistent with ER stress-associated ultrastructural remodeling (Figure 3A,B). We next examined activation of PERK-mediated UPR pathways. Western blot analyses showed time-dependent induction of ATF4 and CHOP, increased phosphorylation of eIF2α, and modulation of PERK levels following ERX-41 treatment across early time points (3–8 h) in both KPL4 and SUM149 cells, confirming robust engagement of ER stress signaling at the protein level (Figure 3C).

### 3.4. Genetic Depletion of LIPA Attenuates ERX-41-Mediated ER Stress Signaling and Cytotoxicity

**Figure 4:**
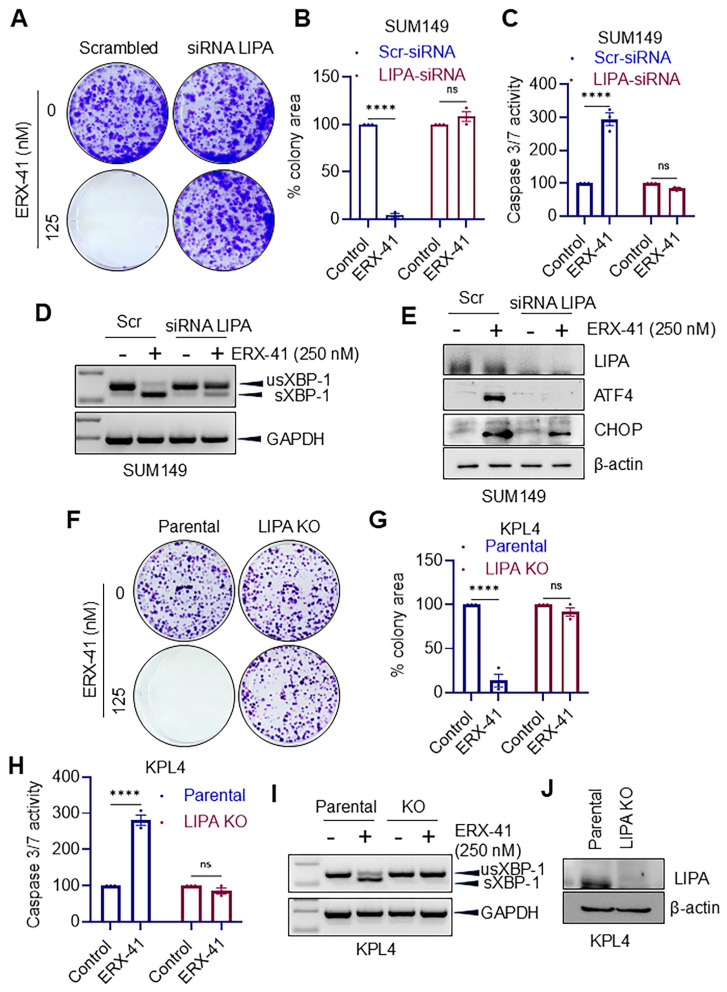
LIPA knockdown or knockout reduces ERX-41 induced ER stress responses and cytotoxic effects. (**A**) Representative images showing colony formation in SUM149 cells transfected with scrambled siRNA or LIPA siRNA and treated with ERX-41 (0 or 125 nM). (**B**) Bar graph showing the percentage colony area in SUM149 cells under the indicated conditions. (**C**) Quantification of caspase-3/7 activity in SUM149 cells transfected with scrambled or LIPA siRNA and treated with ERX-41. (**D**) RT-PCR analysis of *XBP1* splicing in SUM149 cells transfected with scrambled or LIPA siRNA and treated with ERX-41 (250 nM). GAPDH served as a loading control. (**E**) Protein expression of LIPA, ATF4, and CHOP in SUM149 cells transfected with scrambled or LIPA siRNA and treated with ERX-41 (250 nM). β-actin served as a loading control. (**F**) Representative images showing colony formation in parental and LIPA KO KPL4 cells treated with ERX-41. (**G**) Bar graph showing percentage colony area in parental and LIPA KO KPL4 cells under the indicated conditions. (**H**) Quantification of caspase-3/7 activity in parental and LIPA KO KPL4 cells treated with ERX-41. (**I**) RT-PCR analysis of *XBP1* splicing in parental and LIPA-KO KPL4 cells treated with 250 nM ERX-41 (GAPDH loading control). (**J**) LIPA protein levels in parental and LIPA-KO KPL4 cells (β-actin loading control). n = 3; data represent mean ± SEM; **** *p* < 0.0001, ns = not significant.

To test whether ERX-41 response requires LIPA, we knocked down LIPA using siRNA in SUM149 cells and evaluated ERX-41 activity. In scrambled control cells, ERX-41 suppressed colony formation and increased apoptosis-associated caspase-3/7 activity; however, these effects were strongly attenuated in LIPA siRNA conditions (Figure 4A–C). Mechanistically, LIPA depletion reduced ERX-41-induced *XBP1* splicing (Figure 4D) and diminished induction of ATF4 and CHOP protein expression (Figure 4E), indicating that LIPA is required for ERX-41-mediated UPR activation. To further validate specificity, we used KPL4 parental and LIPA knockout (KO) cells. ERX-41 strongly suppressed colony formation and increased caspase-3/7 activity in parental KPL4 cells, whereas LIPA KO cells demonstrated significant resistance to ERX-41 cytotoxicity (Figure 4F–H). Importantly, ERX-41 induced XBP1 splicing and reduced it in LIPA KO cells (Figure 4I), confirming that LIPA is a critical mediator of ERX-41-induced ER stress and cell death in IBC models. LIPA KO was confirmed by immunoblotting (Figure 4J).

### 3.5. ERX-41 Inhibits IBC Tumor Growth In Vivo and Increases ER Stress Marker Expression in Xenografts

**Figure 5:**
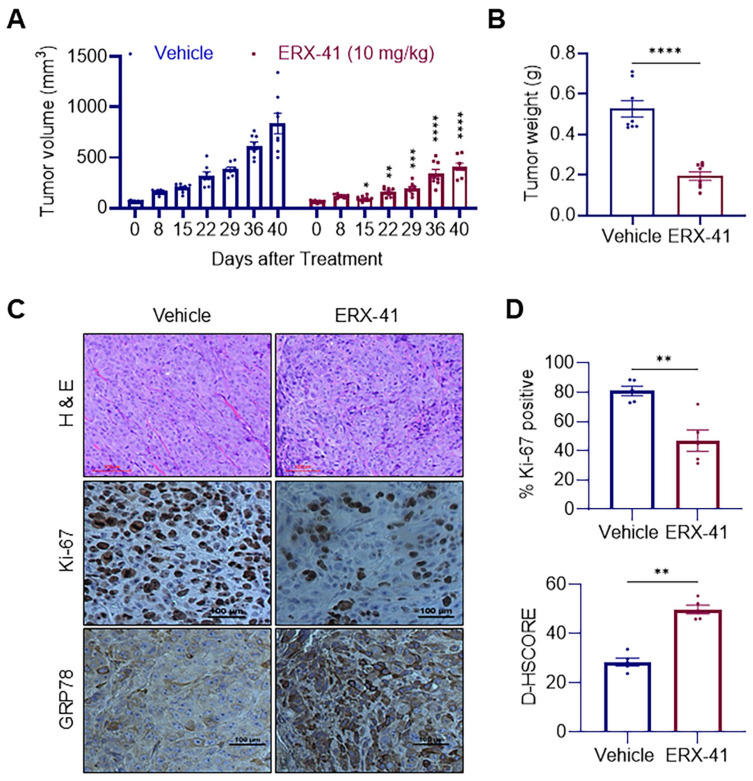
ERX-41 suppresses tumor growth and induces ER stress *in vivo*. (**A**) Tumor volume measurements of KPL4 xenografts in mice treated with vehicle or ERX-41 (10 mg/kg) over 40 days. (**B**) Bar graph showing final tumor weights from vehicle- and ERX-41-treated mice (n = 8). (**C**) Representative tumor sections of H&E staining and IHC staining of Ki-67 and GRP78 from vehicle- and ERX-41-treated mice. (**D**) Percentage of Ki-67^+ve^ cells and GRP78 expression (H-score) in tumor sections from each treatment group. Data represent mean ± SEM; * *p* < 0.05, ** *p* < 0.01, *** *p* < 0.001. **** *p* < 0.0001. Scale bar for GRP78 stained IHC images indicates 100 µm.

To test therapeutic efficacy *in vivo*, we tested the efficacy of ERX-41 in a KPL4 xenograft model. ERX-41 treatment (10 mg/kg) significantly led to the suppression of tumor growth over time compared to vehicle controls (Figure 5A) and reduced final tumor weights (Figure 5B). Immunohistochemical (IHC) analyses of excised tumor sections using Ki-67 staining showed reduced proliferation in ERX-41-treated tumors (Figure 5C,D). In contrast, GRP78 (HSPA5), a canonical ER stress marker, was increased in ERX-41-treated tumors (Figure 5C,D). Together, these *in vivo* results confirm that ERX-41 suppresses IBC tumor progression while simultaneously increasing ER stress signaling in tumor tissue.

## 4. Discussion

IBC remains one of the most lethal forms of BC, characterized by rapid progression, early dissemination, and poor long-term survival despite aggressive multimodal therapy, and treatment resistance remains a major obstacle [5,24]. In this study, we demonstrate that ERX-41, a small molecule that targets LIPA, exerts potent anti-tumor activity in multiple IBC models through activation of ER stress and UPR signaling. Our findings support LIPA as a novel therapeutic vulnerability in IBC and highlight ERX-41 as a promising ER stress-based therapeutic strategy.

IBC is clinically characterized by rapid recurrence and high metastatic potential [2]. Although IBC can arise from any major breast cancer subtype, it is most frequently associated with HER2+ disease or TNBC, reflecting its highly aggressive biological behavior [3]. ERX-41 treatment led to a significant, dose-dependent reduction in cell viability and impaired long-term clonogenic growth, indicating that ERX-41 not only inhibits proliferation but also compromises the sustained survival capacity of IBC cells. We further observed that ERX-41 reduces invasion, suggesting that ERX-41 may suppress not only tumor growth but also metastatic behavior. Collectively, these results indicate that ERX-41 can affect multiple malignant features central to IBC biology.

IBC exists in a high ER stress environment because its aggressively hypoxic, inflamed, and metabolically overloaded tumor microenvironment strongly activates the UPR [25]. ER stress pathway genes are highly expressed in IBC tumor tissues compared to mammary tissues [10]. A key mechanistic contribution of this study is the demonstration that ERX-41 rapidly activates classical UPR signaling pathways in IBC cells. ERX-41 induced early *XBP1* splicing, a hallmark of IRE1α activation, and increased expression of multiple canonical ER stress response genes, including *ATF3, HSPA5/GRP78, CHAC1,* and *HERPUD1*. Importantly, Western blot analyses confirmed robust activation of the PERK-eIF2α arm, including increased p-eIF2α and induction of ATF4 and CHOP, an established pro-apoptotic mediator of unresolved ER stress. This molecular data strongly supports that ERX-41 converts adaptive basal ER stress in IBC into an overwhelming stress response that culminates in apoptosis. The ultrastructural evidence provided by TEM further strengthens this conclusion. We also compared ERX-41 with the standard IBC chemotherapies including doxorubicin and paclitaxel. All three agents significantly reduced IBC cell viability, and importantly, combining ERX-41 with either chemotherapy produced a greater reduction than any single agent alone. These results indicate that ERX-41 may exert additive cytotoxic effects when combined with standard-of-care therapies

LIPA catalyzes the breakdown of cholesteryl esters and triglycerides to produce free fatty acids and cholesterol [26]. Our previous research identified that ERX-41 exerts its activity by targeting LIPA within the endoplasmic reticulum of cancer cells, disrupting ER homeostasis and triggering acute ER stress and activation of the UPR. This leads to accumulation of misfolded proteins, sustained PERK–eIF2α signaling, and ultimately apoptosis when ER stress is not resolved [20]. Importantly, ERX-41 enhances the activity of the ERS system in cancer cells, diminishing its protective functions and inducing apoptosis [20]. Further, ERX-41 selectively kills tumor cells with high basal ER stress, while sparing normal cells which normally have low ER stress [20]. Using LIPA knockdown and knockout approaches, we demonstrate that loss of LIPA significantly attenuates ERX-41-induced apoptosis, suppresses caspase-3/7 activation, reduces inhibition of colony formation, and diminishes UPR signaling outputs in IBC cells. By linking ERX-41 activity directly to LIPA, our work supports LIPA as a druggable upstream regulator of ER homeostasis in IBC cells. Future studies examining detailed molecular mechanisms by which ERX-41 promotes ER stress in IBC cells will help clarify the precise mechanistic bridge between LIPA inhibition and UPR activation.

Prior work has shown that ERX-41 selectively inhibits TNBC cells while sparing non-malignant mammary epithelial cells, demonstrating strong tumor specificity [20]. Comprehensive in vivo toxicity studies revealed no abnormalities in major organs, no changes in body weight during extended dosing, and no effects on plasma cell populations or immunoglobulin secretion [20]. In this study, using a validated dosing regimen and KPL4 xenograft model, ERX-41 significantly reduced tumor volume and final tumor weight, demonstrating clear in vivo activity. Immunohistochemistry of tumor tissues revealed decreased Ki-67 staining, confirming reduced proliferative output in vivo. Further, increased GRP78 expression supports ER stress induction in tumor tissue. However, it is also possible that GRP78 upregulation in tumors can reflect adaptive ER stress. These observations align well with our mechanistic findings in vitro and strengthen confidence that ERX-41 engages ER stress pathways in tumors within a physiological context. Moreover, IBC is often associated with highly proliferative tumors that likely rely on elevated ER capacity for survival under hypoxic and nutrient-stressed microenvironments. This may explain why ER stress induction represents an especially effective approach in IBC.

Our findings suggest several important therapeutic implications. First, ERX-41 may represent a novel class of targeted therapy that induces cell death in IBC cells by exploiting ER stress vulnerability rather than relying on inhibition of canonical oncogenic kinases or hormone signaling. This is attractive because IBC is heterogeneous and often resistant to standard targeted strategies. This work has limitations that should be acknowledged. While we demonstrate ERX-41’s efficacy in in vitro IBC models and in a xenograft model, broader validation in additional patient-derived organoid and xenograft models is warranted to fully capture IBC’s heterogeneity.

## 5. Conclusions

In summary, we identify LIPA as a therapeutic vulnerability in IBC and show that ERX-41 promotes ER stress and UPR, resulting in apoptosis. Target dependence was confirmed using LIPA knockdown/knockout models, and ERX-41 demonstrated potent anti-tumor activity in vivo. These findings provide a streamlined foundation for further preclinical development of ERX-41, including combination-strategy optimization and biomarker-guided applications in IBC.

## Data Availability

All data generated for this study are included within this article.

## Supplementary Materials

The following supporting information can be downloaded at https://www.mdpi.com/article/10.3390/biom16030481/s1. S1: The original image of Western Blot; Table S1: List of primers used for the study.

## Abbreviations

The following abbreviations are used in this manuscript:

IBC	Inflammatory Breast Cancer
BC	Breast Cancer
ER	Endoplasmic Reticulum
ERS	Endoplasmic Reticulum Stress
UPR	Unfolded Protein Response
LIPA	Lysosomal Acid Lipase A
XBP1	X-box Binding Protein 1
CHOP	CCAAT-enhancer-binding Protein Homologous Protein
PERK	PKR-like ER Kinase
ATF4	Activating Transcription Factor 4
IC50	Half Maximal Inhibitory Concentration
RT-qPCR	Real-Time Quantitative Polymerase Chain Reaction
TEM	Transmission Electron Microscopy
IHC	Immunohistochemistry
siRNA	Small Interfering RNA
KO	Knockout
MTT	3-(4,5-Dimethylthiazol-2-yl)-2,5-diphenyltetrazolium Bromide
PBS	Phosphate-Buffered Saline
FBS	Fetal Bovine Serum
DAB	3,3’-Diaminobenzidine
SCID	Severe Combined Immunodeficiency

## References

[1] Woodward W.A. (2015). Inflammatory breast cancer: Unique biological and therapeutic considerations. Lancet Oncol..

[2] Dirix L.Y., Van Dam P., Prové A., Vermeulen P.B. (2006). Inflammatory breast cancer: Current understanding. Curr. Opin. Oncol..

[3] Van Laere S.J., Ueno N.T., Finetti P., Vermeulen P., Lucci A., Robertson F.M., Marsan M., Iwamoto T., Krishnamurthy S., Masuda H. (2013). Uncovering the molecular secrets of inflammatory breast cancer biology: An integrated analysis of three distinct affymetrix gene expression datasets. Clin. Cancer Res..

[4] Tastsoglou S., Karagounis I.V., Miliotis M., Nagandla H., Zambo K.D.A., Liousia M., Ueno N., Maity A., Hatzigeorgiou A.G., Thomas C. (2026). Estrogen receptor β target gene expression reveals novel repressive functions in aggressive breast cancer. NPJ Breast Cancer.

[5] Hance K.W., Anderson W.F., Devesa S.S., Young H.A., Levine P.H. (2005). Trends in inflammatory breast carcinoma incidence and survival: The surveillance, epidemiology, and end results program at the National Cancer Institute. J. Natl. Cancer Inst..

[6] Matro J.M., Li T., Cristofanilli M., Hughes M.E., Ottesen R.A., Weeks J.C., Wong Y.N. (2015). Inflammatory breast cancer management in the national comprehensive cancer network: The disease, recurrence pattern, and outcome. Clin. Breast Cancer.

[7] Ross J.S., Ali S.M., Wang K., Khaira D., Palma N.A., Chmielecki J., Palmer G.A., Morosini D., Elvin J.A., Fernandez S.V. (2015). Comprehensive genomic profiling of inflammatory breast cancer cases reveals a high frequency of clinically relevant genomic alterations. Breast Cancer Res. Treat..

[8] Liang D., Khoonkari M., Avril T., Chevet E., Kruyt F.A.E. (2021). The unfolded protein response as regulator of cancer stemness and differentiation: Mechanisms and implications for cancer therapy. Biochem. Pharmacol..

[9] Bièche I., Lerebours F., Tozlu S., Espie M., Marty M., Lidereau R. (2004). Molecular profiling of inflammatory breast cancer: Identification of a poor-prognosis gene expression signature. Clin. Cancer Res..

[10] Alsterda A., Asha K., Powrozek O., Repak M., Goswami S., Dunn A.M., Memmel H.C., Sharma-Walia N. (2021). Salubrinal Exposes Anticancer Properties in Inflammatory Breast Cancer Cells by Manipulating the Endoplasmic Reticulum Stress Pathway. Front. Oncol..

[11] Guang M.H.Z., Kavanagh E.L., Dunne L.P., Dowling P., Zhang L., Lindsay S., Bazou D., Goh C.Y., Hanley C., Bianchi G. (2019). Targeting Proteotoxic Stress in Cancer: A Review of the Role that Protein Quality Control Pathways Play in Oncogenesis. Cancers.

[12] Zhou R., Wang W., Li B., Li Z., Huang J., Li X. (2025). Endoplasmic Reticulum Stress in Cancer. MedComm.

[13] Chevet E., Hetz C., Samali A. (2015). Endoplasmic reticulum stress-activated cell reprogramming in oncogenesis. Cancer Discov..

[14] Maurel M., McGrath E.P., Mnich K., Healy S., Chevet E., Samali A. (2015). Controlling the unfolded protein response-mediated life and death decisions in cancer. Semin. Cancer Biol..

[15] Avril T., Vauleon E., Chevet E. (2017). Endoplasmic reticulum stress signaling and chemotherapy resistance in solid cancers. Oncogenesis.

[16] He J., Zhou Y., Sun L. (2024). Emerging mechanisms of the unfolded protein response in therapeutic resistance: From chemotherapy to Immunotherapy. Cell Commun. Signal..

[17] Scriven P., Coulson S., Haines R., Balasubramanian S., Cross S., Wyld L. (2009). Activation and clinical significance of the unfolded protein response in breast cancer. Br. J. Cancer.

[18] Almanza A., Carlesso A., Chintha C., Creedican S., Doultsinos D., Leuzzi B., Luis A., McCarthy N., Montibeller L., More S. (2019). Endoplasmic reticulum stress signalling—From basic mechanisms to clinical applications. FEBS J..

[19] Gawlak-Socka S., Kowalczyk E., Wiktorowska-Owczarek A. (2026). Unfolded Protein Response at the Crossroads: Integrating Endoplasmic Reticulum Stress with Cellular Stress Networks. Int. J. Mol. Sci..

[20] Liu X., Viswanadhapalli S., Kumar S., Lee T.K., Moore A., Ma S., Chen L., Hsieh M., Li M., Sareddy G.R. (2022). Targeting LIPA independent of its lipase activity is a therapeutic strategy in solid tumors via induction of endoplasmic reticulum stress. Nat. Cancer.

[21] Viswanadhapalli S., Luo Y., Sareddy G.R., Santhamma B., Zhou M., Li M., Ma S., Sonavane R., Pratap U.P., Altwegg K.A. (2019). EC359-A first-in-class small molecule inhibitor for targeting oncogenic LIFR signaling in triple negative breast cancer. Mol. Cancer Ther..

[22] Schneider C.A., Rasband W.S., Eliceiri K.W. (2012). NIH Image to ImageJ: 25 years of image analysis. Nat. Methods.

[23] Boyce M., Bryant K.F., Jousse C., Long K., Harding H.P., Scheuner D., Kaufman R.J., Ma D., Coen D.M., Ron D. (2005). A selective inhibitor of eIF2alpha dephosphorylation protects cells from ER stress. Science.

[24] Breit C., Sun S. (2025). Updates in the Management of Inflammatory Breast Cancer. Curr. Breast Cancer Rep..

[25] Xu D., Liu Z., Liang M.X., Fei Y.J., Zhang W., Wu Y., Tang J.H. (2022). Endoplasmic reticulum stress targeted therapy for breast cancer. Cell Commun. Signal..

[26] Li F., Zhang H. (2019). Lysosomal Acid Lipase in Lipid Metabolism and Beyond. Arter. Thromb. Vasc. Biol..

