# Supplementary material for: Targeting LIPA with ERX-41 Induces ER Stress and Inhibits Tumor Progression in Inflammatory Breast Cancer
Source: Biomolecules. 2026 Mar 23;16(3):481. doi: 10.3390/biom16030481 (PMC13023744; doi:10.3390/biom16030481)
Supplement: Supplementary file 1 [file biomolecules-16-00481-s001.zip › biomolecules-4154050-supplementary.pdf]

S1. The original image of Western Blot.

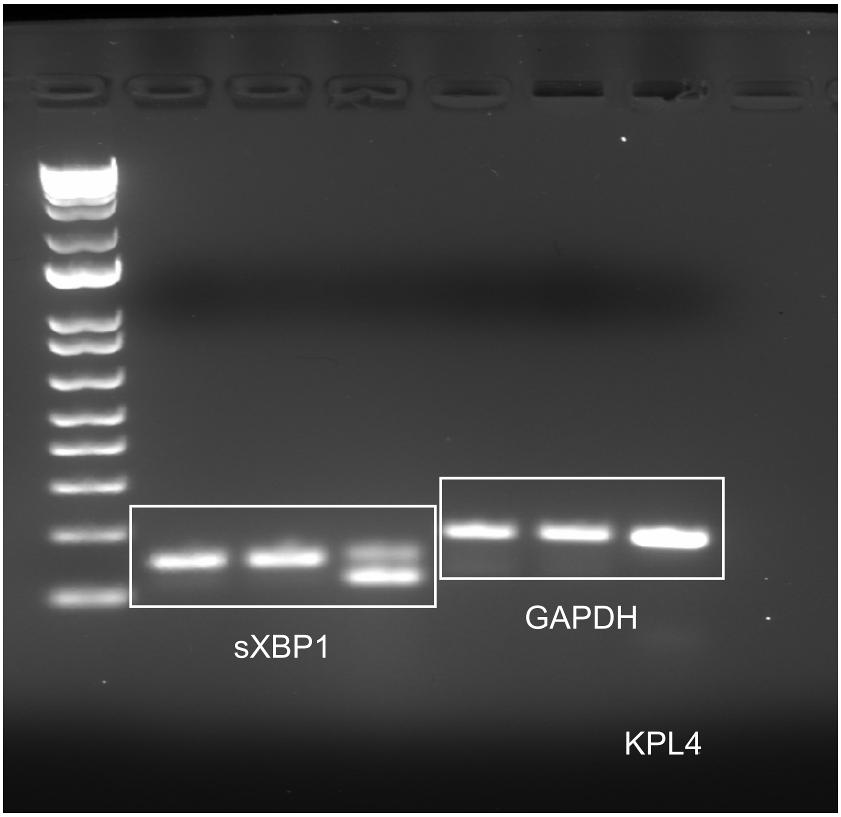

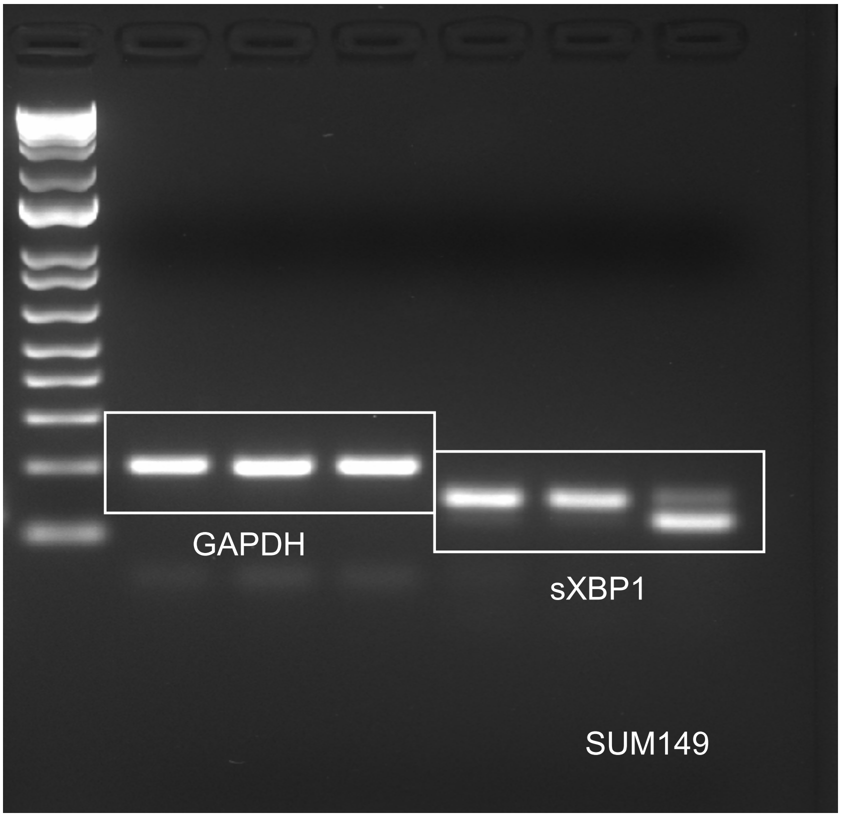

Uncropped agarose gels for Fig. 2C are provided, with the bands used in the final figure indicated by square boxes.

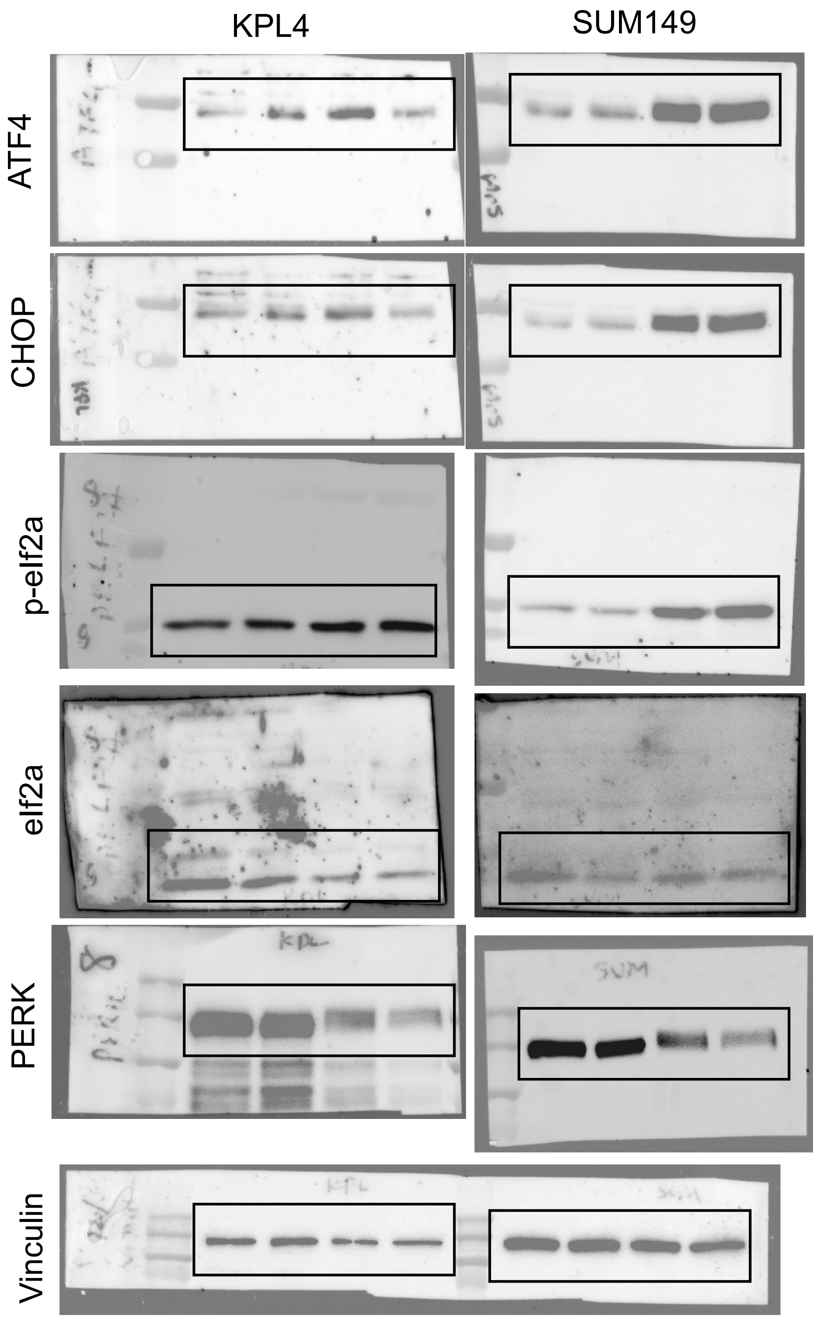

Uncropped Western blots for Fig. 3C are provided, with the bands used in the final figure indicated by square boxes.

4D

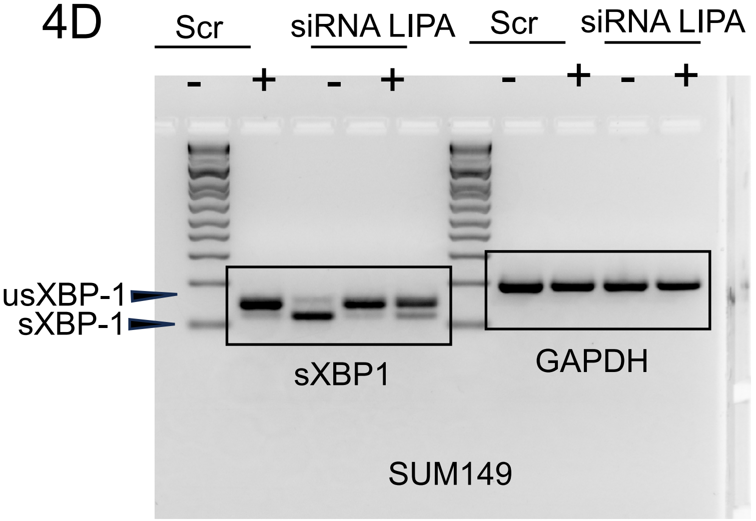

4I

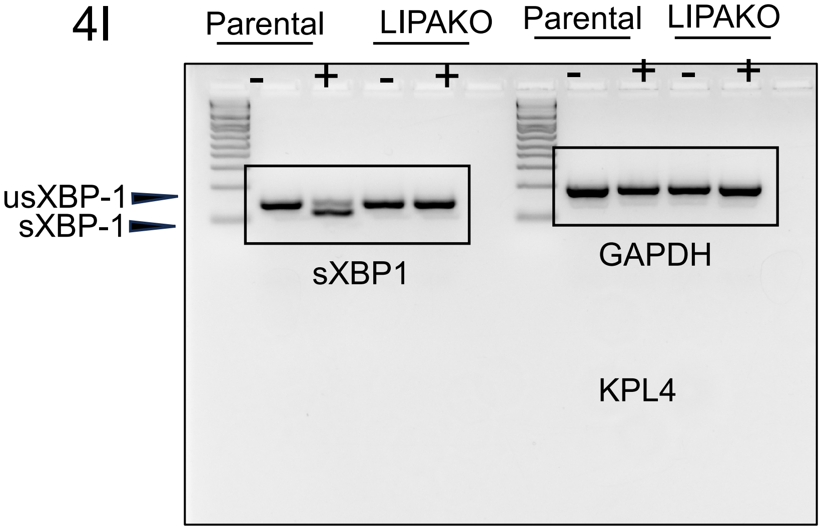

Uncropped agarose gels for Fig. 4D and I are provided, with the bands used in the final figure indicated by square boxes.

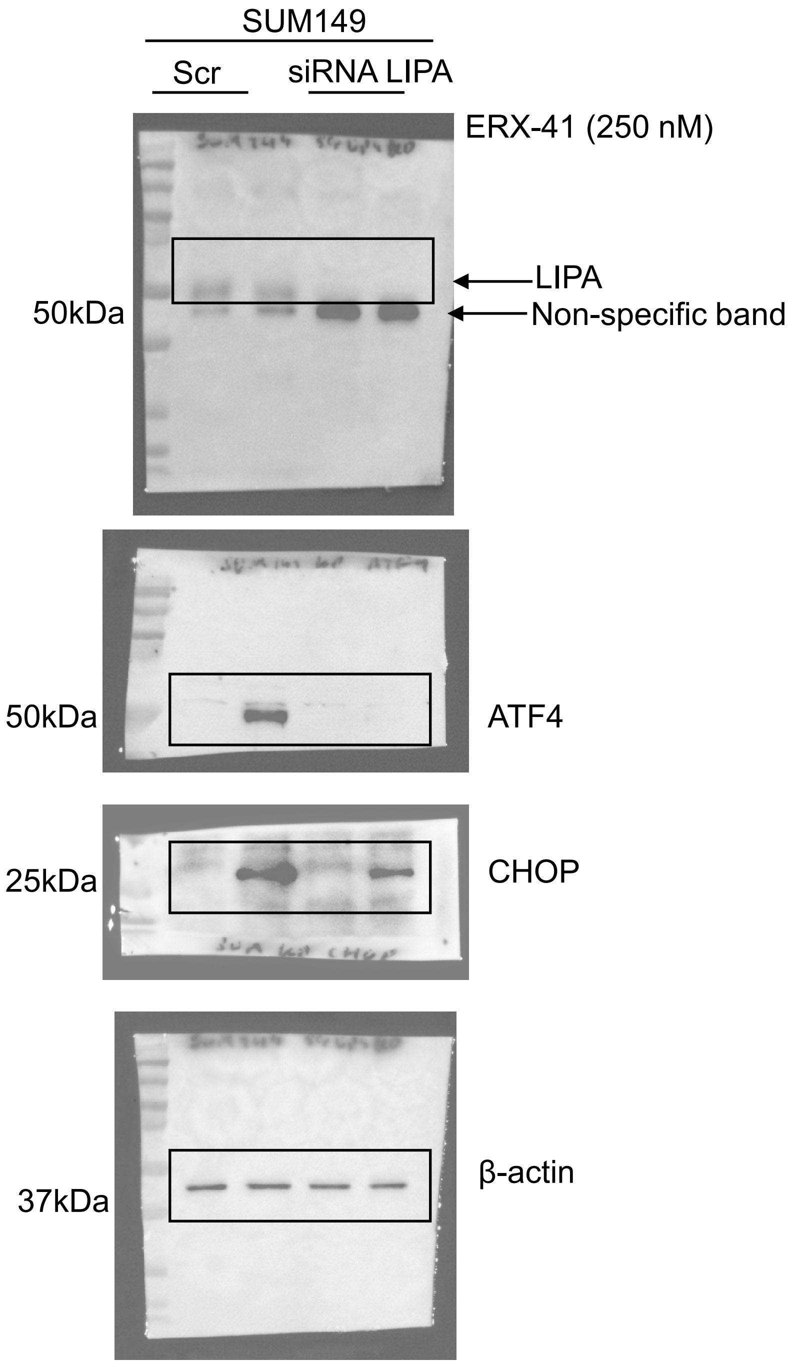

Uncropped Western blots for Fig. 4E are provided, with the bands used in the final figure indicated by square boxes.

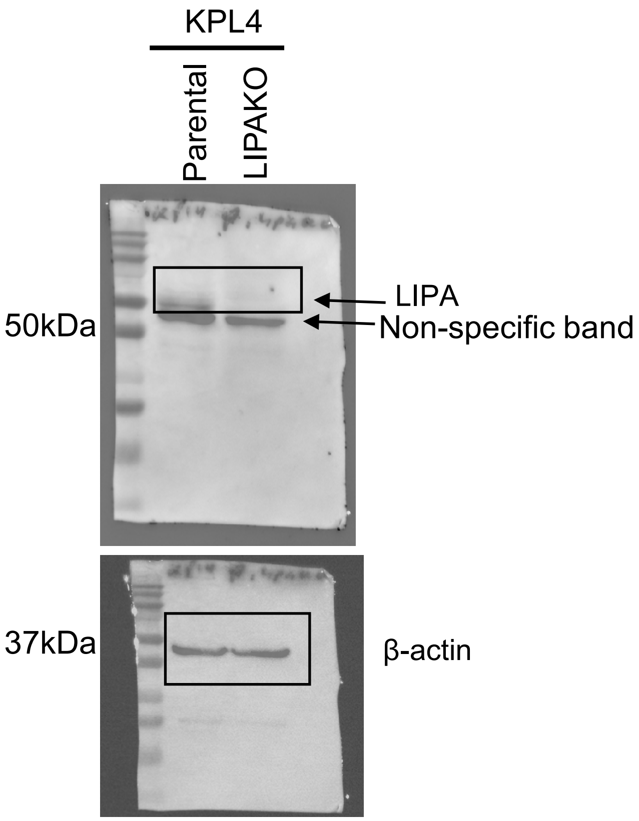

Uncropped Western blots for Fig. 4J are provided, with the bands used in the final figure indicated by square boxes.

<u>Table S1.</u>

List of primers used for the study

Gene name	Forward Primer	Reverse Primer
XBP1	CCCTCCAGAACATCTCCCCAT	ACATGACTGGGTCCAAGTTGT
ATF3	CCTCTGCGCTGGAATCAGTC	TTCTTTCTCGTCGCCTCTTTTT
CHAC1	GAACCCTGGTTACCTGGGC	CGCAGCAAGTATTCAAGGTTGT
HERPUDI	ATGGAGTCCGAGACCGAAC	TTGGTGATCCAACAACAGCTT
STC2	ACAGGTTCGGCTGCATAAGC	GAGGTCCACGTAGGGTTCG
GAPDH	GGAGCGAGATCCCTCCAAAAT	GGCTGTTGTCATACTTCTCATGG
